# Aberrations in the early pregnancy serum metabolic profile in women with prediabetes at two years postpartum

**DOI:** 10.1007/s11306-023-01994-z

**Published:** 2023-03-24

**Authors:** Ella Muhli, Chouaib Benchraka, Mrunalini Lotankar, Noora Houttu, Harri Niinikoski, Leo Lahti, Kirsi Laitinen

**Affiliations:** 1 Institute of Biomedicine, Research Centre for Integrative Physiology and Pharmacology, University of Turku, Turku, 20014 Finland; 2 Department of Obstetrics and Gynecology, University of Turku, Turku, Finland; 3 Department of Computing, Faculty of Technology, University of Turku, Turku, Finland; 4 Department of Pediatrics, Turku University Hospital, Turku, Finland; 5 Functional Foods Forum, University of Turku, Turku, Finland

**Keywords:** Diabetes Mellitus, Type 2, Metabolomics, Pregnancy, Overweight, Obesity

## Abstract

**Introduction:**

Aberrations in circulating metabolites have been associated with diabetes and cardiovascular risk.

**Objectives:**

To investigate if early and late pregnancy serum metabolomic profiles differ in women who develop prediabetes by two years postpartum compared to those who remain normoglycemic.

**Methods:**

An NMR metabolomics platform was used to measure 228 serum metabolite variables from women with pre-pregnancy overweight in early and late pregnancy. Co-abundant groups of metabolites were compared between the women who were (n = 40) or were not (n = 138) prediabetic at two years postpartum. Random Forests classifiers, based on the metabolic profiles, were used to predict the prediabetes status, and correlations of the metabolites to glycemic traits (fasting glucose and insulin, HOMA2-IR and HbA1c) and hsCRP at postpartum were evaluated.

**Results:**

Women with prediabetes had higher concentrations of small HDL particles, total lipids in small HDL, phospholipids in small HDL and free cholesterol in small HDL in early pregnancy (p = 0.029; adj with pre-pregnancy BMI p = 0.094). The small HDL related metabolites also correlated positively with markers of insulin resistance at postpartum. Similar associations were not detected for metabolites in late pregnancy. A Random Forests classifier based on serum metabolites and clinical variables in early pregnancy displayed an acceptable predictive power for the prediabetes status at postpartum (AUROC 0.668).

**Conclusion:**

Elevated serum concentrations of small HDL particles in early pregnancy associate with prediabetes and insulin resistance at two years postpartum. The serum metabolic profile during pregnancy might be used to identify women at increased risk for type 2 diabetes.

**Supplementary Information:**

The online version contains supplementary material available at 10.1007/s11306-023-01994-z.

## Introduction

Maternal metabolism changes during pregnancy to meet the demands of the mother and the feto-placental unit (Lain & Catalano, 2007). Aberrations in these changes are associated with pregnancy complications such as gestational diabetes (GDM) (Kivelä et al., 2021; White et al., 2017), which predisposes the mother herself to subsequent type 2 diabetes and her offspring to obesity in later life (Hod et al., 2015). Prepregnancy overweight is a well-established risk factor for GDM. It has previously been demonstrated, in a cohort of women with overweight, that the serum metabolic profile of women developing GDM differs from those who remain normoglycemic already in early pregnancy (Mokkala et al., 2020). However, thus far, there has been rather little published information on the extent to which the metabolic profile during pregnancy and its potential aberrations influence the onset of diabetes postpartum.

Our working hypothesis was that by undertaking comprehensive examination of metabolic profiles, in addition to traditional metabolic markers, it could be possible to elucidate the associations of circulating metabolites during pregnancy to postpartum metabolic disorders and thus to reveal potential targets for interventions. With respect to the traditional metabolic markers, high third trimester glycated hemoglobin (HbA1c) levels at least 36 mmol/mol (5.4%) have been associated with an increased risk of diabetes mellitus in women with GDM from pregnancy up to five years postpartum (Claesson et al., 2017; Varejão et al., 2021). Elevated high-sensitivity C-reactive protein (hsCRP) levels during mid-pregnancy have also been associated with dysglycemia during the first postpartum year (Durnwald et al., 2018; Ozuguz et al., 2011).

In non-pregnant populations, certain distinct serum metabolites, such as the levels of branched-chain amino acids (BCAAs), as well as those of phenylalanine, glutamate and several lipids, have been associated with an elevated risk of type 2 diabetes (Long et al., 2020) and cardiovascular events (Ruiz-Canela et al., 2017). The association between the serum metabolic profile during pregnancy and a glucose metabolism disorder at postpartum has previously been described in only one publication (Liu et al., 2021); it was reported that fasting serum levels of BCAAs valine, leucine and isoleucine, acylcarnitine C2 and 3-hydroxybutyrate measured at 28 weeks of gestation were associated with prediabetes or type 2 diabetes 10 to 14 years later.

We wanted to investigate the associations of early and late pregnancy serum metabolic profiles to the prediabetes status at two years’ postpartum in an at-risk cohort of women who had overweight before becoming pregnant. We hypothesized that the serum metabolic profiles both in early and late pregnancy would differ between the women with and without prediabetes at two years’ postpartum. The first aim of the study was to investigate the differences in serum metabolites in both early and late pregnancy between the women who later developed prediabetes or remained healthy. We also aimed to investigate if the serum metabolites during pregnancy could be used to predict prediabetes, and furthermore we evaluated the associations of the serum metabolites with glycemic traits at postpartum.

## Methods

### Participants and study design

This study is a follow-up study of women participating in a single-center dietary intervention trial during pregnancy (Pellonperä et al., 2019) (ClinicalTrials.gov: NCT01922791). Briefly, the trial investigated the effect of dietary intervention with fish oil and/or probiotics on maternal and offspring health. The main outcomes were the incidence of GDM and allergy in the offspring. The inclusion criteria were overweight (self-reported prepregnancy BMI ≥ 25 kg/m^2^) and early pregnancy (< 18 weeks of gestation). The exclusion criteria were GDM diagnosed during the current pregnancy, multifetal pregnancy, and metabolic or inflammatory disease, such as type 1 or type 2 diabetes, celiac disease, or inflammatory bowel disease. A total of 439 women were recruited to the intervention trial. Here, we examined 178 women from whom fasting serum samples in early and/or late pregnancy and fasting blood glucose analyzed for diagnosis of prediabetes at two years postpartum were available. The early pregnancy serum metabolomics analysis was available for 174 of the women and the late pregnancy serum metabolomics analysis for 169 of the women. We excluded the women who used GDM medication (metformin, insulin or both; *n* = 10) from the late pregnancy analyses. The women participated in two study visits during pregnancy, in early pregnancy at a mean 13.9 weeks of gestation (SD 2.0 weeks) and in late pregnancy at a mean 35.1 weeks of gestation (SD 0.9 weeks), and in the follow-up visit at two years’ postpartum (mean 2.0 years, SD 0.04 years).

The clinical characteristics of the women were inquired by questionnaires. The intakes of energy and macronutrients were calculated from 3-day food diaries filled in near to the study visits using computerized software (AivoDiet 2.0.2.3, Aivo, Turku, Finland), which utilizes the Finnish Food Composition Database Fineli (Fineli). Blood pressure was measured during the study visits with Omron M5-1 (Intelli™ sense, Omron Matsusaka Co., Ltd, Japan). Height was measured to the nearest 0.1 cm with a wall stadiometer at the early pregnancy study visit. Pre-pregnancy BMI was calculated from self-reported weight, obtained from welfare clinic records, and the height measured in early pregnancy. Mean weekly weight gain between early and late pregnancy was calculated from the weights measured at the study visits.

A standard 2-hour 75-g OGTT for pregnant women was performed (Working group set up by the Finnish Medical Society Duodecim, the Medical Advisory Board of the Finnish Diabetes Association and the Finnish Gynecological Association, 2013) and diagnosis of GDM was based on at least one value at or above the threshold levels: 0 h ≥ 5.3, 1 h ≥ 10.0 and 2 h ≥ 8.6 mmol/l. Prediabetes at two years postpartum was determined according to the criteria issued by the American Diabetes Association (ADA) as fasting plasma glucose from 5.6 to 6.9 mmol/l (American Diabetes Association, 2011). Of the study participants, 40 had prediabetes; two of them reported having type 2 diabetes.

### Blood sampling and analysis

Fasting (9 h minimum) blood samples were drawn from the antecubital vein. The serum was separated and frozen in aliquots at -80 degrees Celsius. The serum metabolic profile was analysed using a high-throughput proton NMR metabolomics platform (Nightingale Health Ltd, Helsinki, Finland) as previously described (Soininen et al., 2015). The platform evaluates 228 metabolites and their ratios, including biomarkers of lipid and glucose metabolism, amino acids, ketone bodies and glycoprotein acetyls (GlycA), a marker of low-grade inflammation. Other sample analyses were assayed in a certified laboratory (Tykslab, the Hospital District of Southwest Finland) immediately after blood sampling. Fasting glucose was measured with an enzymatic method using hexokinase and fasting insulin with an immunoelectrochemiluminometric assay. HbA1c was determined with ion-exchange HPLC. An automated colorimetric immunoassay was used to measure hsCRP. Homeostatic model assessment for insulin resistance (HOMA2-IR) was calculated from fasting glucose and fasting insulin levels (Wallace et al., 2004).

### Statistical analysis

The statistical analysis for the clinical characteristics data of the women was made with IBM SPSS Statistics 28.0 for Windows (IBM SPSS, Chicago, IL, USA). The normality of distributions was visually observed from histograms and evaluated using Shapiro-Wilk’s test. Deviations from normality were assumed when Shapiro-Wilk’s test p < 0.05. The homogeneity of variances was evaluated with Levene’s test (p < 0.05 indicating violation of this assumption). Normally distributed continuous variables are summarized with means and standard deviations and non-normally distributed continuous variables with medians and interquartile ranges. Categorical data are presented as frequencies and percentages.

Differences in the clinical characteristics were evaluated with the Student’s t-test for normally distributed continuous variables and with the Mann-Whitney U-test for non-normally distributed continuous variables. Pearson chi-square test was used for evaluating differences in categorical variables between the groups. Two-sided p-values < 0.05 were considered significant.

Analysis of the metabolomic data was carried out using the R version 4.1.0. In the early pregnancy data, 42 metabolites had missing values; these were imputed using randomly sampled values from the available data of each metabolite independently. Principal Component Analysis (PCA) was done using the *calculatePCA* function from *scater* package (McCarthy et al., 2017), after log_10_ transformation, the data were then scaled to zero mean and unit variance per metabolite, with the functions *log10* and *rowMeans* from the *base* R package (R Core Team, 2021) and *rowSds* from the *matrixStats* package (Bengtsson, 2021).

Co-abundant groups of metabolites were computed by using the original metabolomic data. A dissimilarity matrix was calculated using Spearman correlation, with the help of *cor* function from the *stats R* package (R Core Team, 2021). Then hierarchical clustering was performed with the *hclust from* the *stats* package, using *ward.D2* method and a dissimilarity value cut-off of 0.2 (corresponding to a correlation value of 0.8), *cutree* from *stats* package.

Similar transformation as for the PCA was used to visualize the data for early and late pregnancy as a heatmap, using the *Heatmap* function from the *ComplexHeatmap* package (Gu et al., 2016). The dendrogram visualized with the heatmap was based on the same method to compute the co-abundant groups. We analyzed the 211 prevalent metabolites, with a detection threshold of 0.01 and a prevalence of 10% among the samples; calculated using the *getPrevalentTaxa* function from the *mia* package (Ernst et al., 2022).

In the linear model comparing co-abundant groups of metabolites between the women with and without prediabetes, age, pre-pregnancy BMI and intake of polyunsaturated fatty acids (PUFAs) were chosen as covariates because these differed significantly between the study groups in early pregnancy. The dietary intervention during pregnancy was chosen as a covariate based on prior results (Mokkala et al., 2021). Model 1 included the following covariates: age, intake of PUFAs and the intervention with model 2 including also pre-pregnancy BMI since we wanted to be able to examine metabolites which correlated strongly with BMI. Linear models were carried out using the *lm* function and p-values adjusted with the Benjamini-Hochberg method, *p.adjust* from the *stats* package.

A Random Forests classifier was used to predict prediabetes status based on the metabolomic profiles and covariates separately and combined. This was carried out using the *ranger* package (Wright & Ziegler, 2017) along with cross-validating each model 10 times with the *caret* R package (Kuhn, 2021). To further investigate the predictive power of each model, parallel models with randomly assigned prediabetes status were trained and cross-validated. The performance of each model was reported in terms of area under the receiver operating characteristic curve (AUROC), using the *evalm function* from the *Mleval* (John, 2020).

The association of metabolomic data to glycemic traits was analyzed by Spearman correlation and significance was p-value adjusted with the FDR method, using the *getExperimentCrossCorrelation* from the *mia* package, where three levels of significance were used: 0.2, 0.05 and 0.001. Correlation values and significance were visualized as a heatmap using the *Heatmap* function from the *ComplexHeatmap* package.

## Results

### Clinical characteristics

The majority i.e. 60% of the women were living with overweight and 40% with obesity. Almost a third of the women developed GDM in this current pregnancy; in most cases, it was treated with diet only (Table 1). The women who developed prediabetes by two years postpartum (n = 40) had a higher prepregnancy BMI, were older and were more likely to have had GDM during pregnancy compared to those who did not (n = 138). In addition, their fasting glucose and HbA1c in early pregnancy and fasting glucose, fasting insulin and HOMA2-IR in late pregnancy were higher than those of the women with no prediabetes. There were no significant differences between the groups according to which dietary intervention group they had been assigned in early pregnancy (data not shown). In early pregnancy, the women who later developed prediabetes had greater daily intakes of total fat, monounsaturated fatty acids and PUFAs than those who did not (Online Resource 1).

**Table 1:** Clinical characteristics of the study participants

	All women n = 178	No prediabetes at two years postpartum n = 138	Prediabetes at two years postpartum n = 40	
	n (%)	n (%)	n (%)	P-value
Age in early pregnancy (years; mean, SD)	31.5 (4.6)	31.0 (4.7)	33.0 (4.3)	0.020^a^
Pre-pregnancy BMI (kg/m^2^; median, IQR)	29.0 (26.5‒31.5)	28.4 (26.2‒31.1)	30.5 (27.8‒34.1)	0.011^b^
Ethnicity				1.0^c^
European	175 (98)	135 (98)	40 (100)	
Asian	1 (1)	1 (1)	0 (0)	
Other	2 (1)	2 (1)	0 (0)	
College or university education	116 (65)	94 (68)	22 (55)	0.14^c^
Smoking during pregnancy	6 (3)	4 (3)	2 (5)	0.62^c^
Family history of diabetes or metabolic syndrome	44 (25)	34 (25)	10 (25)	1.0^c^
Primiparous	82 (46)	59 (43)	23 (58)	0.11^c^
Prior GDM	16 (9)	13 (9)	3 (8)	0.77^c^
GDM in the current pregnancy	51 (30)	27 (20)	24 (62)	< 0.001^c^
GDM treatment				< 0.001^c^
diet only	40 (23)	25 (19)	15 (39)	
metformin	6 (4)	2 (2)	4 (10)	
insulin	1 (1)	0 (0)	1 (3)	
insulin + metformin	4 (2)	0 (0)	4 (10)	
Weight gain between early and late pregnancy (kg/week; mean, SD)	0.42 (0.18)	0.43 (0.18)	0.36 (0.16)	0.054^a^
Systolic blood pressure (mmHg)				
early pregnancy (median, IQR)	117.5 (111.3‒125.0)	117.0 (110.5‒125.0)	118.8 (113.8‒124.1)	0.21^b^
late pregnancy (median, IQR)	119.0 (113.1‒128.0)	118.8 (112.9‒127.6)	120.8 (113.1‒132.0)	0.31^b^
Diastolic blood pressure (mmHg)				
early pregnancy (median, IQR)	78.0 (71.5‒83.0)	76.5 (71.0‒82.3)	79.8 (73.5‒84.8)	0.081^b^
late pregnancy (median, IQR)	79.3 (73.1‒86.5)	79.0 (73.4‒85.6)	81.5 (72.0‒91.6)	0.39^b^
Breastfeeding duration (months; median, IQR)	12.0 (6.0‒15.5)	12.1 (6.8‒17.3)	12.0 (3.6‒14.6)	0.36^b^
Fasting glucose (mmol/l)				
early pregnancy (median, IQR)	4.7 (4.5‒5.0)	4.7 (4.5‒4.9)	4.9 (4.6‒5.2)	0.029^b^
late pregnancy (mean, SD)	4.6 (0.4)	4.5 (0.4)	4.8 (0.6)	0.020^a^
2 y postpartum (median, IQR)	5.2 (5.0‒5.5)	5.1 (4.9‒5.3)	5.8 (5.7‒6.2)	< 0.001^b^
HbA1c (mmol/mol)				
early pregnancy (median, IQR)	29.0 (28.0‒31.0)	29.0 (28.0‒31.0)	30.0 (29.0‒32.0)	0.003^b^
2 y postpartum (median, IQR)	32.0 (30.3‒34.0)	32.0 (30.0‒34.0)	35.0 (32.3‒36.0)	< 0.001^b^
Fasting insulin (mU/l)				
early pregnancy (median, IQR)	10.0 (8.0‒14.0)	9.0 (7.0‒14.0)	11.0 (8.0‒13.0)	0.16^b^
late pregnancy (median, IQR)	15.0 (11.0‒20.0)	14.0 (11.0‒19.0)	16.0 (14.8‒22.0)	0.038^b^
2 y postpartum (median, IQR)	11.0 (8.0‒15.0)	10.0 (8.0‒14.0)	14.0 (11.0‒18.8)	< 0.001^b^
HOMA2-IR				
early pregnancy (median, IQR)	1.3 (1.0‒1.8)	1.2 (0.9‒1.8)	1.4 (1.0‒1.7)	0.13^b^
late pregnancy (median, IQR)	1.9 (1.4‒2.4)	1.8 (1.4‒2.4)	2.1 (1.8‒2.8)	0.030^b^
2 y postpartum (median, IQR)	1.5 (1.0‒1.9)	1.3 (1.0‒1.8)	1.9 (1.5‒2.5)	< 0.001^b^
hsCRP (mg/l)				
early pregnancy (median, IQR)	5.6 (3.5‒8.8)	5.5 (3.5‒8.7)	6.3 (3.5‒9.2)	0.48^b^
late pregnancy (median, IQR)	3.9 (2.2‒6.2)	3.9 (2.2‒6.2)	3.6 (2.0‒7.7)	0.95^b^
2 y postpartum (median, IQR)	1.6 (0.7‒3.4)	1.4 (0.7‒3.3)	2.3 (1.1‒4.6)	0.027^b^

GDM, gestational diabetes; HbA1c, glycated hemoglobin; HOMA2-IR, homeostatic model assessment for insulin resistance; hsCRP, high sensitivity C-reactive protein.

^a^Student’s t-test

^b^Mann-Whitney U-test

^c^Chi-square test

### Differences in serum metabolites during pregnancy between the women with and without prediabetes at two years postpartum

**Fig. 1:**
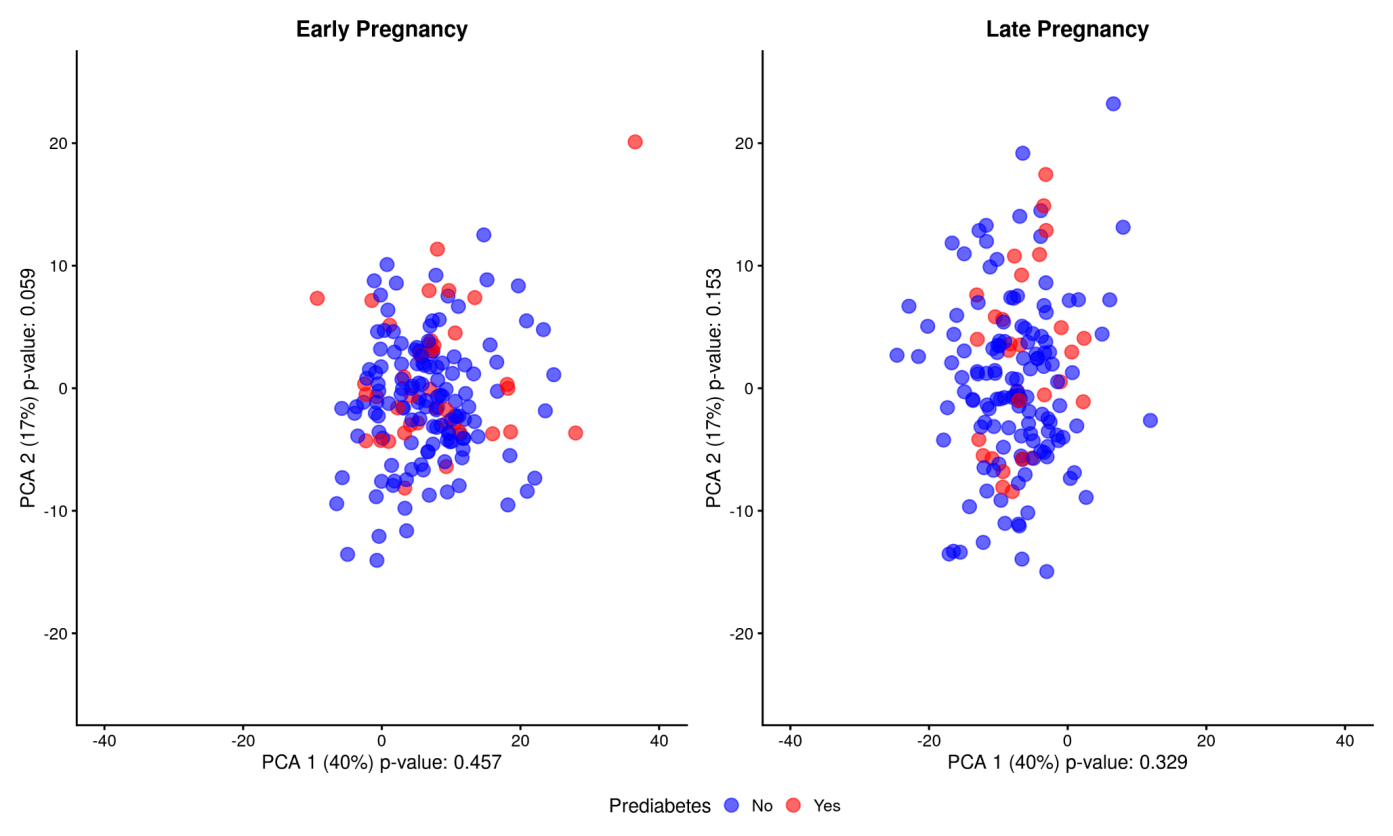
Principal Component Analysis of the serum metabolic profiles of the study participants in early (n = 174) and late pregnancy (n = 159) and prediabetes status at two years postpartum. Blue = no prediabetes and red = prediabetes

The levels and/or ratios of many serum metabolites changed from early to late pregnancy as visualized in Online Resource 2 with the majority of the metabolite concentrations and/or ratios displaying an increase. There was no clear clustering of the metabolites in PCA based on the prediabetes status at two years postpartum in either at early or late pregnancy (Fig. 1).

**Fig. 2:**
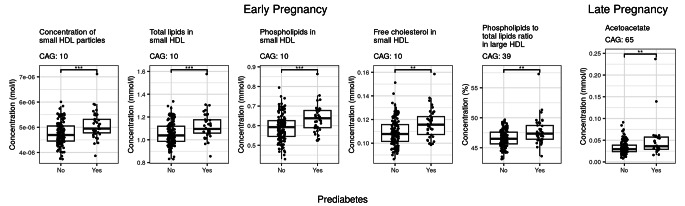
Boxplots of early and late pregnancy serum metabolites, which differed significantly (p < 0.05) between the women with and those without prediabetes at two years postpartum based on a linear model adjusted for age, intervention and intake of polyunsaturated fatty acids. n = 174 in early pregnancy, n = 38 with prediabetes and n = 136 without prediabetes. n = 159 in late pregnancy, n = 29 with prediabetes and n = 130 without prediabetes. The box represents the interquartile range, the line is the median and dots are individual values. ** indicates p-value ≤ 0.01 and *** p-value ≤ 0.001. CAG, co-abundant group of metabolites

Co-abundant groups of serum metabolites in early and late pregnancy were identified using a dissimilarity matrix and hierarchical clustering. Two co-abundant groups of metabolites differed in early pregnancy between the women with and those without prediabetes (Fig. 2). The first group included higher concentrations of small HDL particles, total lipids in small HDL, phospholipids in small HDL and free cholesterol in small HDL in the women with prediabetes compared to the women without prediabetes (p = 0.029, Linear model adjusted for age, intervention and intake of PUFAs). The second group showed a higher phospholipids to total lipids ratio in large HDL particles in the women with prediabetes compared to the women without prediabetes (p = 0.020, Linear model adjusted for age, intervention and intake of PUFAs). When the models were further adjusted for prepregnancy BMI, the associations were attenuated (p = 0.094 and p = 0.070, respectively).

One co-abundant group of metabolites was higher in late pregnancy in the women with prediabetes as compared to women who did not develop this condition (Fig. 2, p = 0.014, Linear model adjusted for age, intervention and intake of PUFAs). The group included only acetoacetate, and it remained significant even after adjusting for the prepregnancy BMI (p = 0.020).

### Prediction of prediabetes status at two years postpartum with serum metabolites during pregnancy

**Fig. 3:**
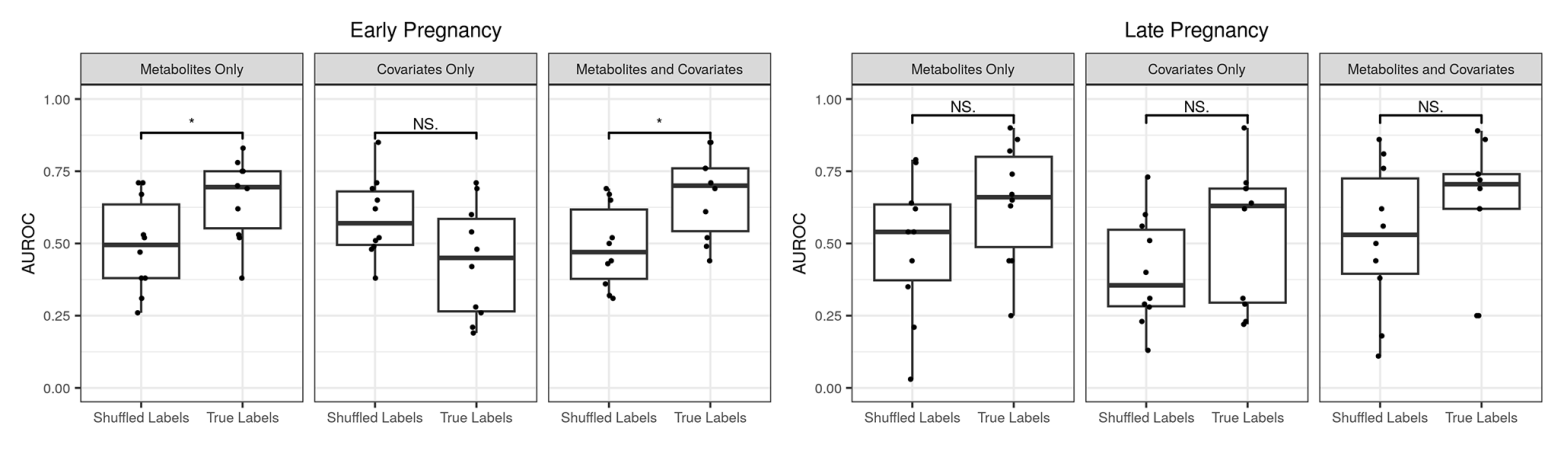
Boxplots of the area under the receiver operating characteristic curve (AUROC) -values of Random Forests models predicting prediabetes status at two years postpartum with serum metabolites, covariates age, intervention, pre-pregnancy BMI and intake of polyunsaturated fatty acids or serum metabolites and covariates. Student’s t-test was used to compare the Random Forests models with true prediabetes status labels to models which assigned the status at random (shuffled labels). n = 38 with prediabetes and n = 136 without prediabetes in early pregnancy, n = 29 with prediabetes and n = 130 without prediabetes in late pregnancy. Box represents interquartile range, line median and dots individual values. * indicates p-value ≤ 0.05. NS, non-significant

We used AUROC of a Random Forests classifier for predicting the prediabetes status at two years’ postpartum. When based on serum metabolites in early pregnancy, the value was 0.655, while it was 0.438 based on only covariates i.e. age, intervention, prepregnancy BMI and intake of PUFAs in early pregnancy and 0.668 when based on both serum metabolites and covariates in early pregnancy. After 10-fold cross-validation, it was observed that the classifier based on serum metabolites and covariates performed the best (Fig. 3). The following five serum metabolites and covariates were the most important features in the classifier; glycerol, cholesterol esters to total lipids ratio in very large HDL particles, acetoacetate, free cholesterol to total lipids ratio in large HDL and age. Both the classifier based on serum metabolites and the classifier based on serum metabolites and covariates performed significantly better than those classifiers which randomly assigned the prediabetes status.

The AUROC of a Random Forests classifier predicting the prediabetes status at two years postpartum based on serum metabolites in late pregnancy was 0.64, which was a similar value as that obtained with the classifier based on both serum metabolites and covariates in late pregnancy (AUROC 0.638). The AUROC value of the classifier based on covariates only in late pregnancy was somewhat lower (AUROC 0.53). None of the classifiers differed significantly from the classifiers which assigned the prediabetes status at random (Fig. 3).

### The correlations between serum metabolites during pregnancy and glycemic traits at two years postpartum

**Fig. 4:**
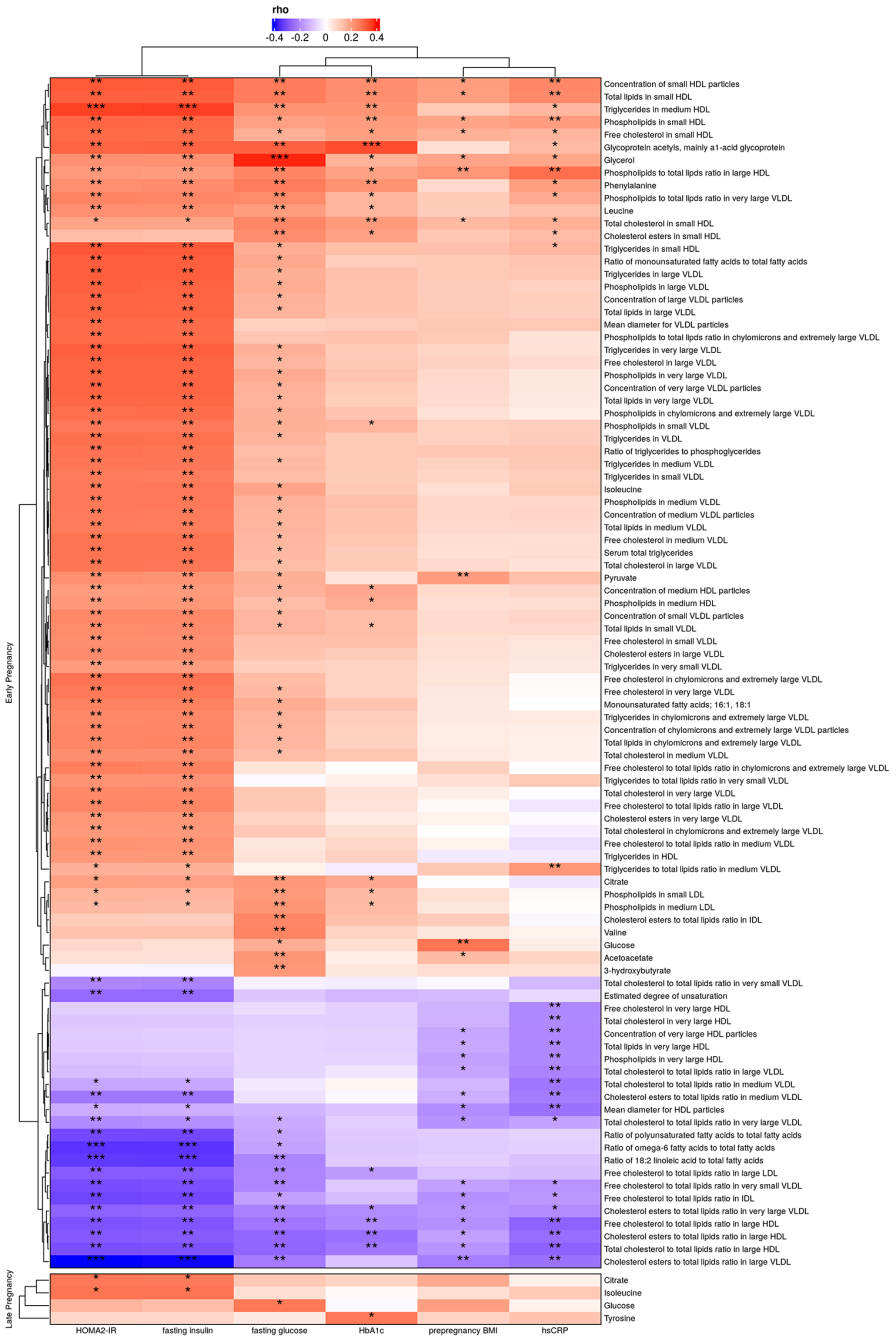
Heatmaps of FDR-adjusted Spearman correlations between serum metabolites in early or late pregnancy and glycemic traits at two years’ postpartum. n = 174 in early pregnancy and n = 159 in late pregnancy. * indicates FDR ≤ 0.2, ** FDR ≤ 0.05 and *** FDR ≤ 0.001. HOMA2-IR, homeostatic model assessment for insulin resistance; HbA1c, glycated hemoglobin; hsCRP, high sensitivity C-reactive protein

FDR-adjusted Spearman correlations were used to investigate the associations of serum metabolites in early and late pregnancy to glycemic traits (fasting glucose and insulin, HOMA2-IR and HbA1c) and hsCRP at two years postpartum (Fig. 4). The concentrations of small HDL particles, total lipids in small HDL, phospholipids in small HDL and free cholesterol in small HDL in early pregnancy correlated positively with all of the glycemic traits, in addition to pre-pregnancy BMI, at two years postpartum. There were highly significant (FDR ≤ 0.001) positive correlations between triglycerides in medium size HDL particles in early pregnancy and fasting insulin and HOMA2-IR. The inflammatory marker GlycA correlated positively with all of the glycemic traits, especially with HbA1c at FDR-level ≤ 0.001. Along with many VLDL related variables, the levels of BCAAs (i.e. leucine and isoleucine) and monounsaturated fatty acids correlated positively with fasting insulin and HOMA2-IR. The concentrations of valine, leucine and two ketone bodies i.e. acetoacetate and 3-hydroxybutyrate correlated positively with that of fasting glucose. Glycerol had a highly significant positive correlation with fasting glucose. Highly significant negative correlations were detected between the ratios of n-6 fatty acids to total fatty acids and linoleic acid to total fatty acids and fasting insulin and HOMA2-IR.

Of the serum metabolites in late pregnancy, the concentration of isoleucine correlated positively at FDR-level ≤ 0.2 with fasting insulin and HOMA2-IR at two years postpartum (Fig. 4). Similarly, the glucose concentration correlated positively with fasting glucose, citrate with fasting insulin and HOMA2-IR and tyrosine with HbA1c.

## Discussion

In this study, we demonstrated that women who developed prediabetes by two years postpartum had higher serum concentrations of small HDL particles, total lipids in small HDL, phospholipids in small HDL and free cholesterol in small HDL in early pregnancy and higher serum concentrations of acetoacetate in late pregnancy as compared to women who did not develop prediabetes. The small HDL related variables also correlated positively with HbA1c and markers of insulin resistance at two years postpartum.

We detected elevated serum levels of small HDL particles in early pregnancy in the women who developed prediabetes by two years postpartum, although the association was dependent on the pre-pregnancy BMI. In a large previous study of non-pregnant women, higher levels of small HDL particles and a smaller HDL particle size were associated with incident type 2 diabetes during a follow-up of 13 years (Mora et al., 2010). Similarly, a larger HDL particle size has been associated with a decreased risk of type 2 diabetes in young adults (Ahola-Olli et al., 2019). In addition to type 2 diabetes, elevated concentrations of small HDL particles have been linked with a risk of cardiovascular disease (Kontush, 2015). During early pregnancy, higher concentrations of small HDL particles have been shown to predict GDM (Mokkala et al., 2020). Thus, it appears that high levels of small HDL particles are associated with an increased cardiometabolic risk at different stages of the lifecycle. Therefore it is not unreasonable that we detected similar metabolic features in pregnant women prior to the onset of GDM and prediabetes, as GDM is a known risk factor for type 2 diabetes (Bellamy et al., 2009). Further studies are warranted to clarify if elevated serum levels of small HDL particles during pregnancy predict the onset of type 2 diabetes, especially in women affected by GDM, thus identifying a possible high-risk group in need of targeted screening and interventions to prevent the onset of diabetes. A dietary intervention would represent a feasible approach to exert an impact on metabolism. Indeed, in the same cohort as studied here, dietary supplementation with fish oil and probiotics during pregnancy induced favorable alterations in serum lipid variables, although the alterations were less evident in women with GDM (Mokkala et al., 2021). Since only 40 women developed prediabetes by two years postpartum, we did not investigate the impact of the dietary intervention here. The incidence of prediabetes did not differ between the dietary intervention groups.

In addition to the levels of small HDL particles, the levels of two BCAAs leucine and isoleucine, the aromatic amino acid phenylalanine and an inflammatory marker GlycA in early pregnancy correlated positively with markers of insulin resistance at two years postpartum. BCAAs are among the best-established metabolic markers for type 2 diabetes (Long et al., 2020). Higher levels of leucine, isoleucine, phenylalanine and GlycA have been associated with an increased risk for type 2 diabetes in young adults (Ahola-Olli et al., 2019). Liu et al. examined women at 28 weeks of gestation and observed an association between elevated BCAA levels and prediabetes or type 2 diabetes 10 to 14 years later (Liu et al., 2021). Elevated serum levels of leucine, isoleucine and GlycA during pregnancy have also been frequently associated with GDM (Kivelä et al., 2021; Mokkala et al., 2020; White et al., 2017). Recently it has been suggested that higher BCAA and GlycA levels point to a susceptibility to develop type 2 diabetes already decades before the onset of the disease (Bell et al., 2020) and that in fact insulin resistance causally affects BCAA metabolism (Mahendran et al., 2017; Wang et al., 2017). As reviewed recently (White et al., 2021), obesity and insulin resistance *per se* may increase circulating BCAA levels, which in turn contribute to the development of cardiometabolic diseases.

The levels of acetoacetate, a ketone body, were elevated in late pregnancy in the women who later developed prediabetes. It was also the third most important predictor of future prediabetes in early pregnancy in our Random Forests classifier. In addition to this finding, Liu et al. detected an association between another ketone body, 3-hydroxybutyrate measured at 28 weeks of gestation, and postpartum prediabetes or type 2 diabetes (Liu et al., 2021). In a cohort of middle-aged men, elevated fasting levels of acetoacetate were associated with incident type 2 diabetes during a 5-year follow-up and with impaired insulin secretion rather than insulin resistance (Mahendran et al., 2013). Increased levels of acetoacetate have also been detected in women with GDM prior to and at the time of the diagnosis (White et al., 2017). It is evident that the rate of ketogenesis in the liver is regulated by multiple factors; ketogenic substrates include fatty acids and amino acids, especially leucine (Puchalska & Crawford, 2017), which could link elevated acetoacetate levels to increased BCAA metabolism.

Based on our results, serum metabolites in early pregnancy could predict future prediabetes when combined in a model with clinical variables. A similar predictive power has been observed in a previous study examining women at 28 weeks of gestation (Liu et al., 2021), but the improvement in the prediction compared to traditional clinical factors alone was minimal. Compared to our Random Forests classifier and the clinical variables based on baseline differences between the prediabetes groups, Liu et al. used a wider range of clinical factors in their model, such as a family history of diabetes, parity and OGTT results during pregnancy. They also included fewer metabolites based on Lasso regression analysis in their model. In our model, glycerol was the most important predictor. In addition to fasting glucose at two years postpartum, it correlated with fasting insulin and HOMA2-IR. Circulating levels of glycerol and fatty acids are elevated by excessive lipolysis in adipose tissue, a feature encountered in individuals with obesity and insulin resistance. Glycerol and fatty acids in turn may promote insulin resistance in skeletal muscle and liver. (Bódis & Roden, 2018)

This study has several strengths. We examined an at-risk group of women for metabolic disorders and had a standardized protocol recording multiple clinical variables from early pregnancy until two years postpartum. All blood samples were collected in the fasting state. The comprehensive collection of background data allowed us to include potential confounding factors into the statistical analyses. Nonetheless, if there had been a larger number of study participants with prediabetes, this could have made it possible to reveal other serum metabolites associated with prediabetes in addition to those detected here, and thus future trials with a larger number of participants are called for in order to verify our findings. In addition, the maternal BMI value may have influenced the associations of metabolites to prediabetes, although we included pre-pregnancy BMI as a covariate in the analyses. Clearly it would be informative to examine the associations also in individuals with normal weight. The fact that the study cohort included women from a high-income European country might somewhat limit the generalization of the results, but the mean age and parity of the study participants correlate well with values currently observed in the Finnish population (Official Statistics of Finland, Perinatal statistics. THL., 2021).

## Conclusion

Aberrant serum metabolic profile was detected in early pregnancy in women who developed prediabetes by two years postpartum, namely elevated serum concentrations of small HDL particles, and increased total lipids, phospholipids and free cholesterol in small HDL particles. The association seems to depend on pre-pregnancy BMI. Together with traditional clinical markers, the assessment of the serum metabolic profile in early pregnancy could potentially be used to predict future prediabetes risk. Future studies will be needed to clarify whether the metabolic features detected here reveal an at-risk group of women who would benefit from interventions to prevent type 2 diabetes during and after pregnancy.

## Electronic supplementary material

Below is the link to the electronic supplementary material.

Supplementary Material 1

Supplementary Material 2

## Data Availability

The data sets are not available due to the fact that they contain information that could compromise the privacy and consent of the participants. The source code for the analyses is available online (10.5281/zenodo.7743884).

## Abbreviations

AUROC: Area under the receiver operating characteristic curve
BCAA: Branched-chain amino acid
GDM: Gestational diabetes
GlycA: Glycoprotein acetyls
HbA1c: Glycated hemoglobin
HOMA2-IR: Homeostatic model assessment for insulin resistance
hsCRP: High-sensitivity C-reactive protein
PCA: Principal Component Analysis
PUFA: Polyunsaturated fatty acid
