# Supplementary material for: Aberrations in the early pregnancy serum metabolic profile in women with prediabetes at two years postpartum
Source: Metabolomics. 2023 Mar 24;19(4):20. doi: 10.1007/s11306-023-01994-z (PMC10038958; doi:10.1007/s11306-023-01994-z)
Supplement: Supplementary file 1 — Supplementary Material 1 [file 11306_2023_1994_MOESM1_ESM.pdf]

Ella Muhli, Chouaib Benchraka, Mrunalini Lotankar, Noora Houttu, Harri Niinikoski, Leo Lahti, Kirsi Laitinen. **Aberrations in the early pregnancy serum metabolic profile in women with prediabetes at two years postpartum**, Metabolomics

Corresponding author: Ella Muhli; elromu@utu.fi; Institute of Biomedicine, Research Centre for Integrative Physiology and Pharmacology, University of Turku, 20014 Turku, Finland

***Supplementary Table** Daily nutrient intakes of the study participants in early and late pregnancy calculated from 3-day food diaries*

		All women n=178	No prediabetes n=138	Prediabetes n=40	
		mean (SD)	mean (SD)	mean (SD)	P-value
Energy, kJ					
	early pregnancy	8450 (2028)	8294 (1907)	8968 (2339)	0.065[a]
	late pregnancy	8288 (1978)	8311 (20149)	8195 (1826)	0.77[a]
Carbohydrates, g					
	early pregnancy	224.5 (60.5)	222.7 (58.8)	230.2 (66.2)	0.50[a]
	late pregnancy	214.8 (58.4)	216.1 (56.3)	209.5 (67.3)	0.58[a]
Protein, g					
	early pregnancy	80.2 (20.6)	78.9 (20.4)	84.4 (20.8)	0.14[a]
	late pregnancy (median, IQR)	77.3 (64.4–90.1)	76.9 (64.1–90.7)	78.6 (66.1–87.6)	0.96[b]
Fat, g					
	early pregnancy (median, IQR)	80.9 (64.6–98.5)	79.6 (63.1–97.2)	87.9 (71.7–109.6)	0.038[b]
	late pregnancy (median, IQR)	82.6 (64.5–97.2)	80.6 (64.4–98.3)	88.1 (67.2–96.0)	0.59[b]
Dietary fiber, g					
	early pregnancy (median, IQR)	20.8 (15.4–26.2)	20.8 (16.2–26.3)	20.1 (14.6–24.9)	0.61[b]
	late pregnancy (median, IQR)	19.1 (15.1–24.2)	19.1 (15.3–23.6)	17.9 (14.6–26.0)	0.61[b]
Saturated fatty acids, g					
	early pregnancy (median, IQR)	28.7 (22.6–36.0)	27.4 (22.4–35.4)	31.5 (22.9–41.2)	0.16[b]
	late pregnancy (median, IQR)	30.4 (22.6–37.5)	30.0 (22.1–37.7)	33.1 (24.4–37.1)	0.42[b]
Monounsaturated fatty acids, g					
	early pregnancy (median, IQR)	26.6 (21.0–34.2)	26.2 (20.7–33.8)	29.4 (25.4–39.1)	0.043[b]
	late pregnancy (median, IQR)	27.7 (22.0–34.4)	27.6 (21.5–34.8)	28.9 (22.1–33.0)	0.72[b]
Polyunsaturated fatty acids, g					
	early pregnancy (median, IQR)	12.6 (8.8–16.3)	12.1 (8.7–15.6)	14.9 (10.0–18.1)	0.032[b]
	late pregnancy (median, IQR)	12.3 (9.3–15.2)	12.3 (9.1–15.7)	12.3 (10.1–13.9)	0.81[b]
Carbohydrates, E%					
	early pregnancy	45.3 (6.3)	45.8 (6.3)	43.7 (6.5)	0.077[a]
	late pregnancy (median, IQR)	45.0 (39.8–49.0)	45.3 (40.0–48.8)	43.3 (38.3–49.6)	0.64[b]

Protein, E%					
	early pregnancy (median, IQR)	16.0 (14.0–18.3)	16.0 (14.1–18.2)	16.0 (13.8–18.6)	0.96[b]
	late pregnancy (median, IQR)	16.5 (14.4–18.3)	16.4 (14.5–18.2)	16.8 (14.2–19.0)	0.51[b]
Fat, E%					
	early pregnancy	36.0 (6.4)	35.5 (6.2)	37.6 (6.7)	0.070[a]
	late pregnancy	37.1 (6.6)	36.9 (6.3)	38.0 (7.7)	0.38[a]
Saturated fatty acids, E%					
	early pregnancy	13.1 (3.0)	13.0 (3.0)	13.3 (3.1)	0.61[a]
	late pregnancy (median, IQR)	13.3 (11.1–16.3)	13.2 (11.1–15.9)	14.2 (11.5–17.6)	0.32[b]
Monounsaturated fatty acids, E%					
	early pregnancy (median, IQR)	12.1 (10.3–13.8)	12.1 (10.3–13.7)	12.8 (10.4–14.9)	0.18[b]
	late pregnancy (median, IQR)	12.4 (11.0–14.4)	12.2 (11.0–14.1)	13.0 (10.7–15.2)	0.38[b]
Polyunsaturated fatty acids, E%					
	early pregnancy (median, IQR)	5.6 (4.6–6.6)	5.4 (4.5–6.5)	5.9 (4.9–6.8)	0.099[b]
	late pregnancy (median, IQR)	5.4 (4.5–6.7)	5.4 (4.5–6.8)	5.4 (4.5–6.6)	0.96[b]

[a]Student's t-test
[b]Mann-Whitney U-test
