# Supplementary figures and images for: Aberrations in the early pregnancy serum metabolic profile in women with prediabetes at two years postpartum
Source: Metabolomics. 2023 Mar 24;19(4):20. doi: 10.1007/s11306-023-01994-z (PMC10038958; doi:10.1007/s11306-023-01994-z)

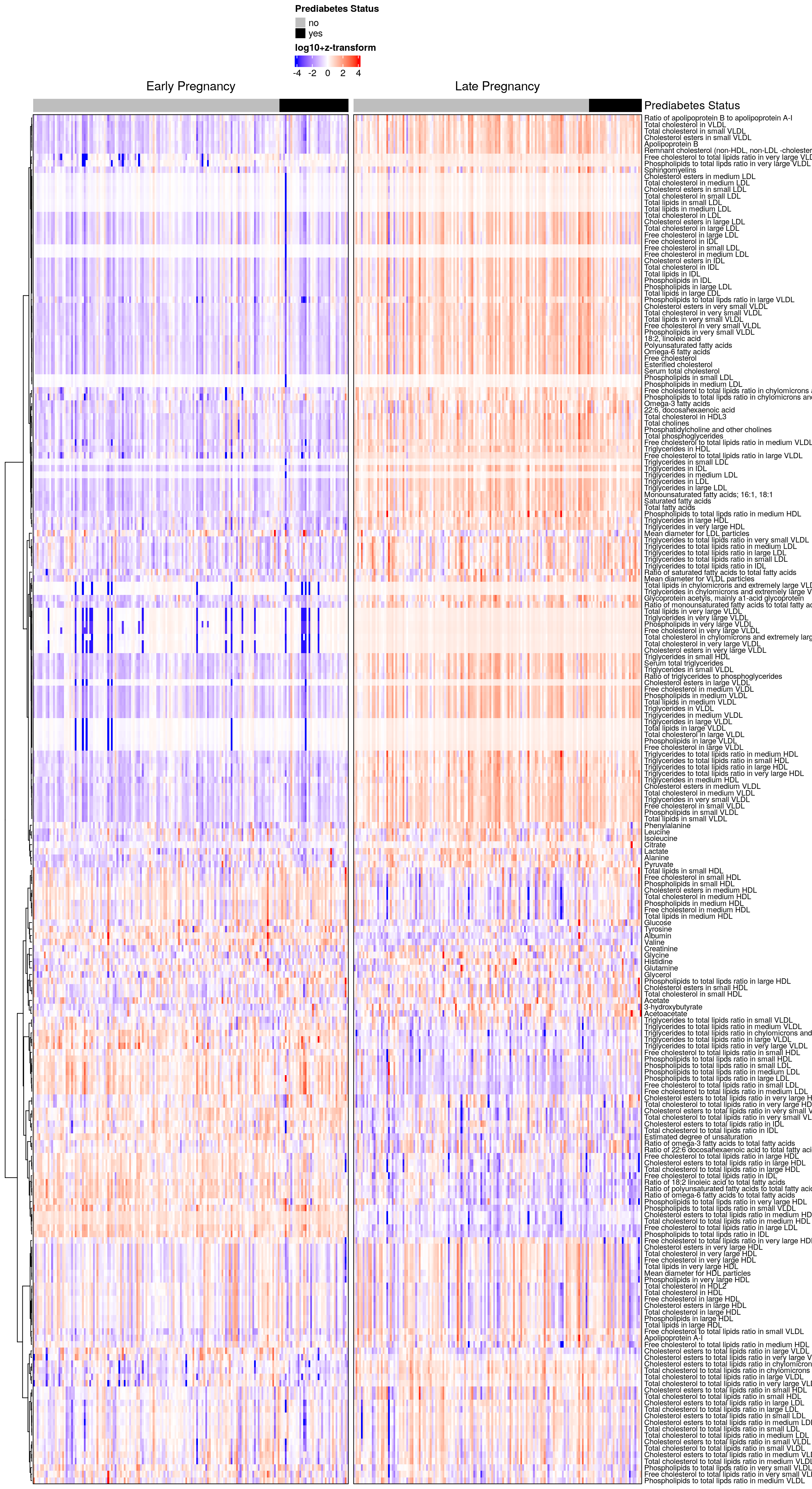

Supplement: Supplementary file 2 — Supplementary Material 2 [file 11306_2023_1994_MOESM2_ESM.tif]
